# 

**Published:** 1878-10

**Authors:** 


					﻿OCTOBEB, 1878.
TWENTY-FIFTH YEAR—No. 298.
HALL’S
Journal of Health.
PUBLISHED AT 74 FOURTH AVENUE,
TxTETW YORK.
E. H. GIBBS, A.M., M.D., Editor.
Medical Office—139 East Eighth Street, Near Broadway.
OFFICE HOURS—FROM 10 A.M. TO 3 F.M.
AGENTS FOR THE TRADE:
AMERICAN NEWS COMPANY AM NEW YORK NEWS COMPANY.
Terms, $1.50 a Year, or $1.00 for Eight Months. Postage paid.
SINGLE NUMBER, 15 CENTS.
Couch & Johnston, Printers, 11 Frankfort Street, New York.
				

